# BALB/c mice infection with hybrid *Leishmania* (*V.*) *guyanensis*/*L.* (*V.*) *shawi* showed an intermediate virulence profile compared to parental species infections

**DOI:** 10.3389/fcimb.2025.1648268

**Published:** 2025-09-11

**Authors:** Ana Carolina Stocco Lima, Thaise Yumie Tomokane, Gabriela Fernandes Rodrigues, Larissa dos Santos Alcântara, Marliane Batista Campos, Maíra Pombo, Márcia Dalastra Laurenti, Vania Lucia Ribeiro da Matta, Lucile Maria Floeter-Winter, Carlos Eduardo Pereira Corbett, Fernando Tobias Silveira, Cláudia Maria de Castro Gomes

**Affiliations:** ^1^ Laboratório de Patologia de Moléstias Infecciosas (LIM50), Departamento de Patologia, Faculdade de Medicina, Universidade de Sao Paulo, Sao Paulo, Brazil; ^2^ Ministério da Saúde, Secretaria de Vigilância em Saúde, Instituto Evandro Chagas, Seção de Parasitologia, Ananindeua, PA, Brazil; ^3^ Departamento de Ciências Biológicas e da Saúde, Universidade Federal do Amapá, Macapá, Amapá, Brazil; ^4^ Instituto de Biociências, Universidade de São Paulo, São Paulo, SP, Brazil; ^5^ Núcleo de Medicina Tropical, Universidade Federal do Pará, Belém, Pará, Brazil

**Keywords:** cutaneous leishmaniasis, *L. (V.) guyanensis*, *L. (V.) shawi*, biological behavior, hybrid parasite

## Abstract

**Introduction:**

Hybridization events within the genus *Leishmania* have been documented; however, their impact on the infection dynamics of hybrids remains poorly understood. In this study, we compared the infection dynamics caused by a hybrid parasite, *Leishmania* (*Viannia*) *guyanensis*/*Leishmania* (*Viannia*) *shawi*, with those caused by its parental species, *Leishmania* (*Viannia*) *guyanensis* and *Leishmania* (*Viannia*) *shawi*, in BALB/c mice.

**Methods:**

Balb/c mice were inoculated with stationary-phase promastigote forms of each parasite. Lesion development and parasite load were monitored longitudinally, and cytokine production was assessed at 35 days post-infection (PI).

**Results:**

The infection with the hybrid parasite induced a more rapid and evident progression, attaining its largest dimension between days 14 and 28 days PI, followed by regression. In contrast, infection with *L*. (*V*.) *guyanensis* resulted in a continuous increase in swelling, whereas *L*. (*V*.) *shawi* caused only mild swelling. Parasite loads in skin and lymph nodes were comparable across groups, though the hybrid parasite exhibited a significant increase in parasite burden from day 35 PI onwards.

**Discussion:**

The immunologic response of hybrid parasite infection was associated with reduced gamma interferon (IFN-γ) and elevated interleukin 4 (IL-4) production compared to parental species and controls (P < 0.05), with no significant differences observed in interleukin 12 (IL-12p40) or interleukin 10 (IL-10). Infection with *L*. (*V*.) *guyanensis* led to decreased IFN-γ in lymph nodes and increased IL-4 production in both skin and lymph nodes, whereas *L*. (*V*.) *shawi* infection did not significantly alter cytokine profiles.

**Conclusion:**

Together, these findings provide important insights into the distinct biological behavior of the *Leishmania* hybrid parasite and its parental species, underscoring the relevance of hybridization in shaping host-parasite interactions and advancing our understanding of leishmaniasis within complex eco-epidemiological settings.

## 1 Introduction

Leishmaniases are among the six major worldwide diseases according to the World Health Organization (2022). In Brazil, cutaneous leishmaniasis occurs throughout the national territory. The great diversity of species characteristic of the Amazon Rainforest also extends to vectors, reservoirs, and *Leishmania* species, resulting in a complex eco-epidemiological scenario with overlapping niches, thus complicating the understanding of the parasite’s biology (Nolder et al., 2007). In the context of leishmaniasis in the Brazilian Amazon, Santarém municipality, Pará State, is particularly prominent due to the occurrence of both cutaneous and visceral forms, as well as the record of five out of the seven species causing cutaneous leishmaniasis in Brazil, and the presence of subpopulations of *Leishmania* (*Viannia*) *shawi* and seven hybrid lineages between *L*. (*V*.) *guyanensis* and *L*. (*V*.) *shawi* (Jennings et al., 2014; Lima et al., 2021).

Reproduction and population structure of organisms in the genus *Leishmania* is predominantly clonal (Tibayrenc, 1995; Tibayrenc and Ayala, 2012; Tomasini et al., 2014). With the advent of increasingly sophisticated molecular tools, knowledge regarding the reproductive mechanisms and the generation of diversity in the genus *Leishmania* has been continually evolving (Tibayrenc et al., 1991; Akopyants et al., 2009; Sterkers et al., 2011; Rogers et al., 2014).

Although sexual reproduction in the genus *Leishmania* is well documented, the frequency and contribution of sexual events to population dynamics are still widely debated (Bastien et al., 1992; Akopyants et al., 2009; Rougeron et al., 2009; Tibayrenc and Ayala, 2013). Moreover, little is known about how these events contribute to the phenotypic properties of hybrids, including the expression of factors that influence infectivity, virulence, and transmissibility, for example (Torrico et al., 1999). *In vivo* infection experiments, for example, have shown that hybrids can display enhanced infectivity and higher parasite burdens (Kelly et al., 1991; Delgado et al., 1997; Nolder et al., 2007; Romano et al., 2014). Hybrid parasites have also been reported to gain advantages in colonization and development within insect vectors, thereby increasing their transmission potential (Volf et al., 2007).

Given the limited availability of isolated hybrid parasites for studies investigating the impacts of natural genetic recombination between different *Leishmania* species on parasite biology and disease manifestation, this study aimed to characterize the infection profile induced by a *Leishmania* (*Viannia*) *guyanensis*/*Leishmania* (*Viannia*) *shawi* hybrid in susceptible mouse strains and to compare it with the profiles produced by each parental species.

## 2 Materials and methods

### 2.1 Animals

Four-week-old female BALB/c mice were obtained from Bioterism Center of the School of Medicine, University of São Paulo. The animals were housed in the Experimental Animal Facility of the Institute of Tropical Medicine of São Paulo, Brazil under controlled ventilation with food and water provided *ad libitum*. All animal protocols in this publication were approved by the “Research Ethics Committee of FMUSP” under the authorization protocol number 048/12. The tests were made according to n°11.794 law of 08/03/2008.

### 2.2 Parasites

Promastigotes of *L*. (*V*.) *shawi* (MCEB/BR/1984/M8408), *L*. (*V*.) *guyanensis* (MHOM/BR/1775/M4147), and the hybrid *L*. (*V*.) *guyanensis*/*L*. (*V*.) *shawi* (MHOM/BR/1996/M19672) were kindly provided by the Leishmaniasis Laboratory “Prof. Dr. Ralph Lainson” of the Evandro Chagas Institute (Surveillance Secretariat of Health, Ministry of Health), Pará State, Brazil.

### 2.3 Experimental BALB/c infection

Stationary-promastigotes were inoculated at 10^6^ cells in sterile PBS by footpad (Lima et al., 1997). All tests were carried out with parasites in recent passage after isolation of the vertebrate host. Four experimental groups were used: Three experimental groups were inoculated with *L*. (*V*.) *guyanensis* (M4147), *L*. (*V*.) *shawi* (M8408) and *L*. (*V*.) *guyanensis*/*L*. (*V*.) *shawi* (M19672) parasites, each one containing thirty-five BALB/c animals. One control group consisted of five animals that were inoculated with a sterile PBS solution.

### 2.4 Evolution of the swelling in BALB/c mice paw

Swelling progression was monitored weekly for 49 days post-infection (PI) by measuring footpad with a micrometer. Swelling was expressed as the difference between the mean footpad swelling of infected mice and that of the uninfected control group.

### 2.5 Skin and lymph node parasite load

The parasitic load of infected animals was analyzed using the limiting dilution assay in the skin and lymph node samples at 48 hours, 7, 14, 21, 30 and 60 days PI (Lima et al., 1997). At each time point, five mice were sedated with xylazine and ketamine and euthanized by CO_2_ inhalation. Lesion skin and the popliteal draining lymph node were collected, weighed, and homogenized in 1 mL of Schneider’s medium supplemented with 10 % heat-inactivated fetal bovine serum (FBS-Gibco™), 10 microgram per ml (µg/mL) of gentamicin (Gibco^tm)^ and ampicillin (1 000 IU- Gibco™). From the maceration of these biopsies in culture medium, 11 serial dilutions were performed at the ratio of 1:3 in quadruplicate. The final wells served as negative controls. Plates were sealed and incubated at 25°C for 10 days to allow differentiation of amastigotes into promastigotes. Wells were examined under an inverted microscope on days 5 and 10 of incubation to verify the maximum titration in which viable promastigotes were found. Then, the promastigotes/weight ratio of each tissue fragment was calculated to estimate the parasite load (number of parasites/grams of host tissue), according to the equation:


$$\begin{array}{c} Parasite\;load=\\ \;\frac{reciprocal\;of\;the\;largest\;positive\;bond\;\times material\;dilution\;\times \left(\frac{homogenate\;volume}{volume\;of\;the\;1^{st}\;well}\right)}{Organ\;weight} \end{array}$$

### 2.6 Temporal dynamics of parasite load

The evolution of infection, assessed through variations in parasite load in skin lesions and lymph nodes over time, was analyzed by multiple linear regression using R software to compare the infection profiles among different parasite groups.

### 2.7 Cytokines production

A quantitative analysis of the production of cytokines in the skin and lymph node of BALB/c mice in experimental infection by the hybrid *L*. (*V.*) *guyanensis*/*L*. (*V.*) *shawi* (M19672) was carried out, as well as a comparison with the production of the cytokines during infection by parental strains. Interferon gamma (IFN-γ), interleukin 12 subunit 40 (IL-12p40), interleukin 4 (IL-4) and interleukin 10 (IL-10) cytokines were quantified using the Milliplex ^®^MAG Mouse Cytokine Magnetic beads Panel Kit (MYCTOMAG-70 K-Merck-Millipore). The cytokines were quantified in the skin and lymph nodes of mice at 35 days after infection (PI) for a comparative analysis of the profile of the immune response established during infection by these studied species.

### 2.8 Statistical analysis

Statistical analyses were conducted in GraphPad Prism v5 (GraphPad Software, San Diego, CA, USA). Data are reported as arithmetic mean ± standard error. The data were compared across the three experimental groups at each time point using two-way analysis of variance (ANOVA), followed by Bonferroni’s *post hoc* test for multiple comparisons. To analyze parasite load, preliminary global and pairwise comparisons of means were performed across all experimental factors (time, strain, and tissue) using ANOVA with Bonferroni correction. To further characterize infection dynamics, temporal regression analyses were conducted. Given the exponential progression of parasite load, data were log_10_+1 transformed to stabilize variances and linearize relationships. The transformed data were then subjected to stepwise backward multiple regression based on the Akaike Information Criterion (AIC), starting from a full linear model that included all three-way interactions. This combined analytical approach allowed for the integration of both static (mean-based) and dynamic (time-resolved) perspectives, enhancing the robustness of the findings.

## 3 Results

### 3.1 Evolution of swelling in BALB/c mice paw

**Figure 1:**
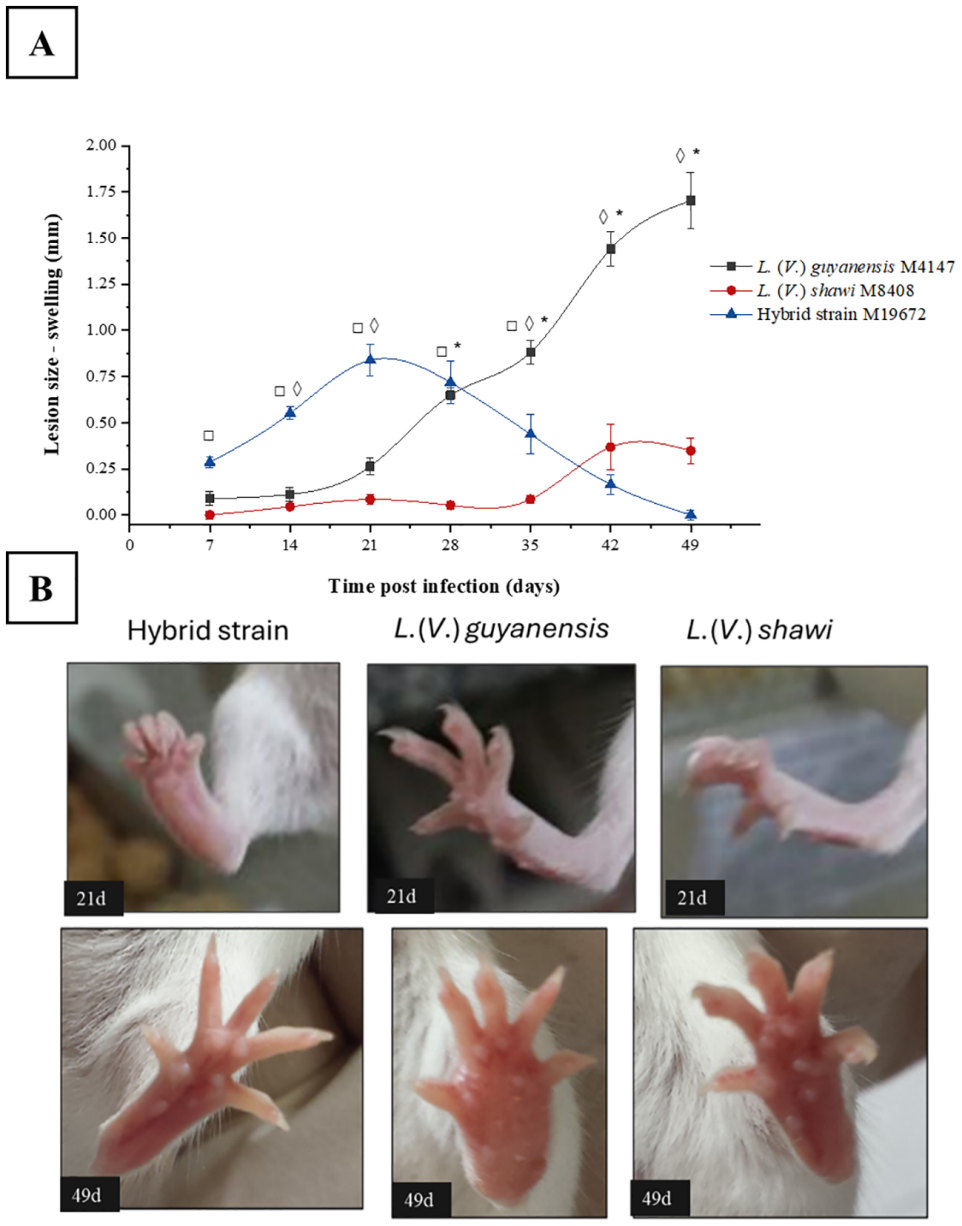
Swelling progression of the BALB/c mouse foot caused by the hybrid strain M19672, *L*. (*V*.) *guyanensis* (M4147) and *L*. (*V*.) *shawi* (M8408). **(A)**, Swelling. Symbols (*, ◊, □): indicate statistical significance (P < 0.05) among the different analyzed groups: * = *L*. (*V*.) *guyanensis* × *L*. (*V*.) *shawi*; ◊: *L*. (*V*.) *guyanensis* × M19672; □ = *L*. (*V*.) *shawi* × M19672. Data presented consists of the mean values ± standard error. **(B)**, BALB/c swelling mouse foot at 21- and 49-days PI.

The evolution of swelling caused by experimental infection with the hybrid *L*. (*V.*) *guyanensis*/*L*. (*V.*) *shawi* and the parental species *L*. (*V*.) *guyanensis* and *L*. (*V*.) *shawi* in BALB/c mice was monitored weekly for 49 days after infection. The infection caused by the hybrid parasite showed a continuous and rapid evolution with a discreet peak between 14- and 28-days post infection (PI), when it reached a mean increase of 0.75 mm (**Figures 1A, B**). From the 28^th^ day PI, there was a regression in the swelling of the footpad, which practically disappeared after 49 days of infection. The swelling caused by *L*. (*V*.) *guyanensis* began its progression as early as 21 days PI and continued to increase until the 49^th^ day, reaching approximately 2 mm in diameter. (**Figures 1A, B**). Infection by *L*. (*V*.) *shawi* did not cause apparent damage until the 28^th^ day PI. From the 35^th^ day PI, the mice showed a slight increase in the swelling of the footpad until the 42^nd^ day PI, which remained similar until the last time analyzed (**Figures 1A, B**).

### 3.2 Evolution of parasite load over time

**Figure 2:**
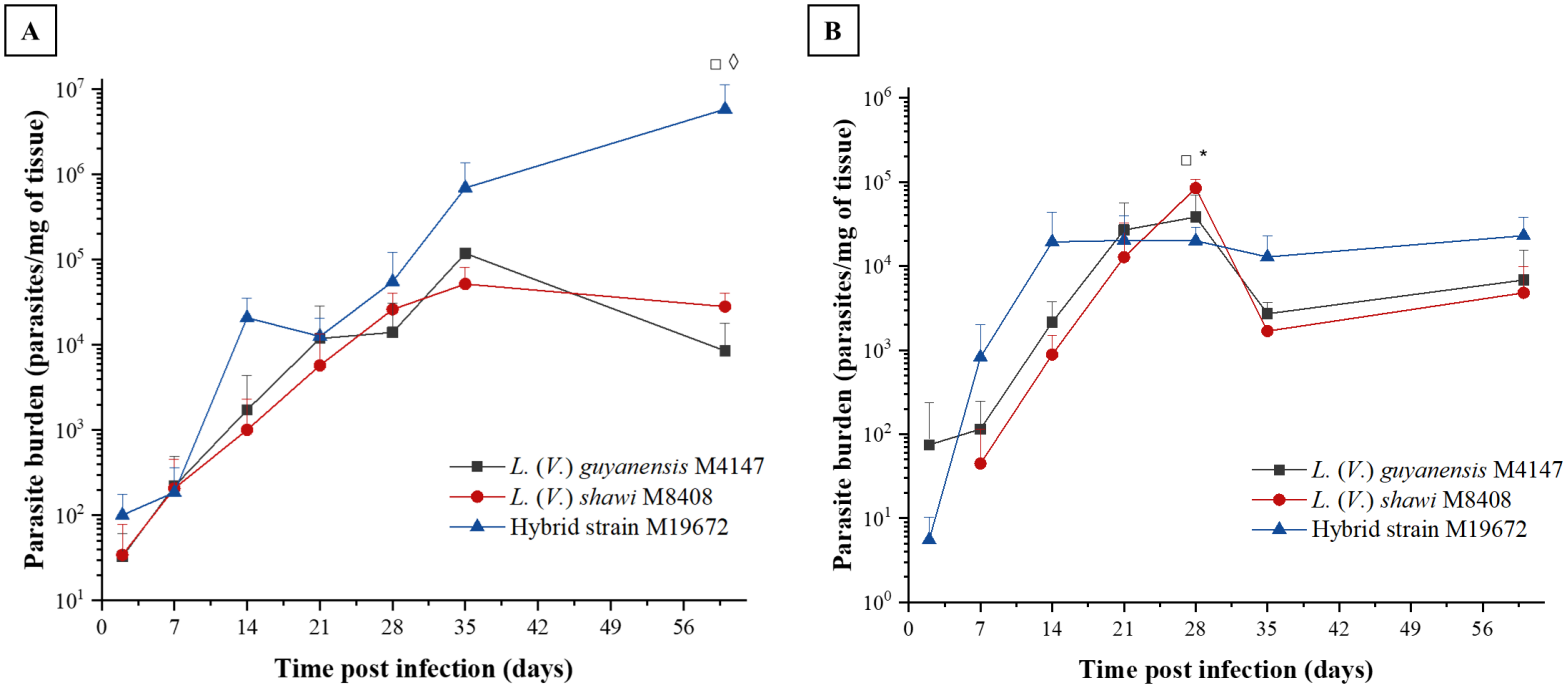
Parasite load in BALB/c mouse skin **(A)** and lymph node **(B)** caused by infection with hybrid parasite M19672, *L*. (*V*.) *guyanensis* (M4147), and *L*. (*V*.) *shawi* (M8408). The mice were inoculated with 10^6^ parasites in the stationary phase in the hind footpad. Symbols (*, ◊, □): indicate statistical significance (P < 0.05) among the different analyzed groups: ◊: *L*. (*V*.) *guyanensis* × hybrid (M19672); □ = *L*. (*V*.) *shawi* × M19672; * = *L*. (*V*.) *guyanensis* × *L*. (*V*.) *shawi*. The data presented are the mean values ± standard error.

The skin parasite load caused by the parental *L*. (*V*.) *shawi* and *L*. (*V*.) *guyanensis* showed a similar profile, showing continuous growth until the 21^st^ day PI and remained stable until the 60^th^ day PI (**Figure 2A**). The skin parasite load caused by the hybrid showed a continuous evolution over time, becoming significantly higher at 60 days after infection with a parasite load of 5.85 × 10^6^ parasites per mg of tissue, compared with 2.81 × 10^4^ for *L*. (*V*.) *shawi* and 8.52 × 10^3^ for *L*. (*V*.) *guyanensis* (p < 0.01). In the lymph node, the parasite load was similar between the hybrid parasite and its parental species over time. Only at 28 days did *L*. (*V*.) *shawi* present a parasite load of 8.45 × 10^4^ parasites/mg of tissue, which was higher (p < 0.01) than the parasite loads of *L*. (*V*.) *guyanensis* (3.83 × 10^4^ parasites/mg of tissue) and the hybrid strain M19672 (2 × 10^4^ parasites/mg of tissue) as observed in **Figure 2B**.

### 3.3 Temporal dynamics of parasite load

**Figure 3:**
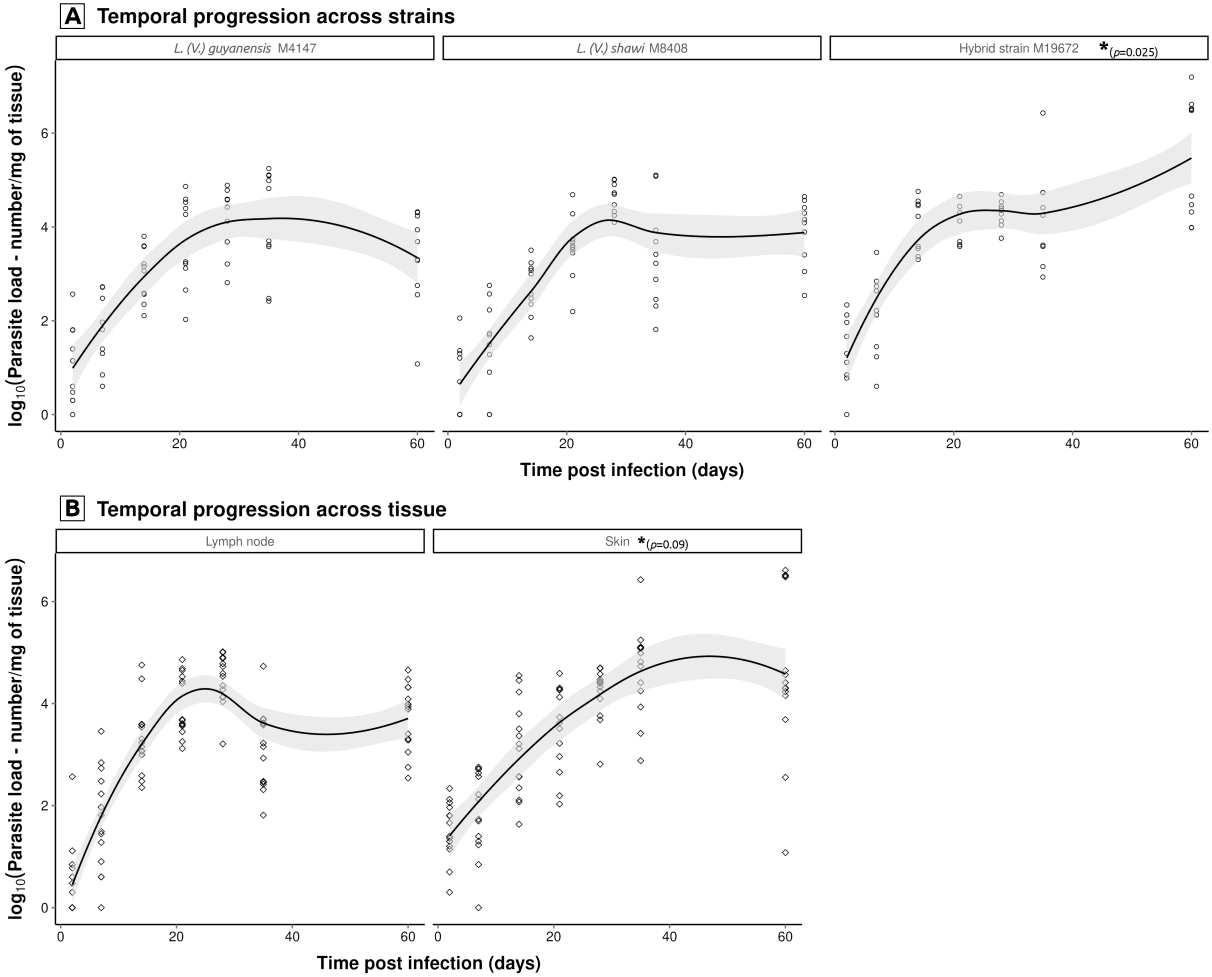
Significant temporal interactions modulating *Leishmania* parasite load dynamics. Multiple regression modeling of log_10_-transformed (log10 + 1) parasite loads (promastigotes/mg tissue) identified two key interaction effects: **(A)** strain × time (hybrid strain M19672 load exhibited significantly higher proliferation intensity (p<0.05) than both *L*. (*V*.) *guyanensis* (M4147) and *L*. (*V*.) *shawi* (M8408), which showed similar kinetic, and **(B)** tissue × time (skin demonstrated elevated parasite loads compared to lymph node, regardless *Leishmania* strain). Non-parametric LOESS curves (span = 0.75) with 95% confidence bands illustrate overall trends. Statistically significant pairwise differences, indicated by asterisks and respective p-values, were identified through linear modeling of log_10_-transformed data.

Model selection revealed that parasite load dynamics are governed by independent time-strain and time-tissue interactions, while excluding non-significant three-way (time × tissue × strain) and direct tissue-strain effects. These results demonstrate a strain-specific proliferation pattern that remains consistent across tissues, with the hybrid strain M19672 showing the highest levels, followed by *L*. (*V*.) *shawi* M8408, and *L*. (*V*.) *guyanensis* M4147 (**Figure 3A**). The tissue-specific progression rates were marginally significantly higher in skin (p = 0.09) than in lymph nodes across all strains (**Figure 3B**), as detected in the temporal progression between tissues, it gains relevance considering that the model was based on log-transformed data. This final model explains 48.3% of variance (adj. r² = 0.46, F(7,191) = 25.46, p < 0.001), with residual error = 1.107 log_10_ units.

### 3.4 Cytokine profile in the skin and lymph node of BALB/c mice infected with hybrid *L*. (*V*.) *guyanensis*/*L*. (*V*.) *shawi* and its parental species

**Figure 4:**
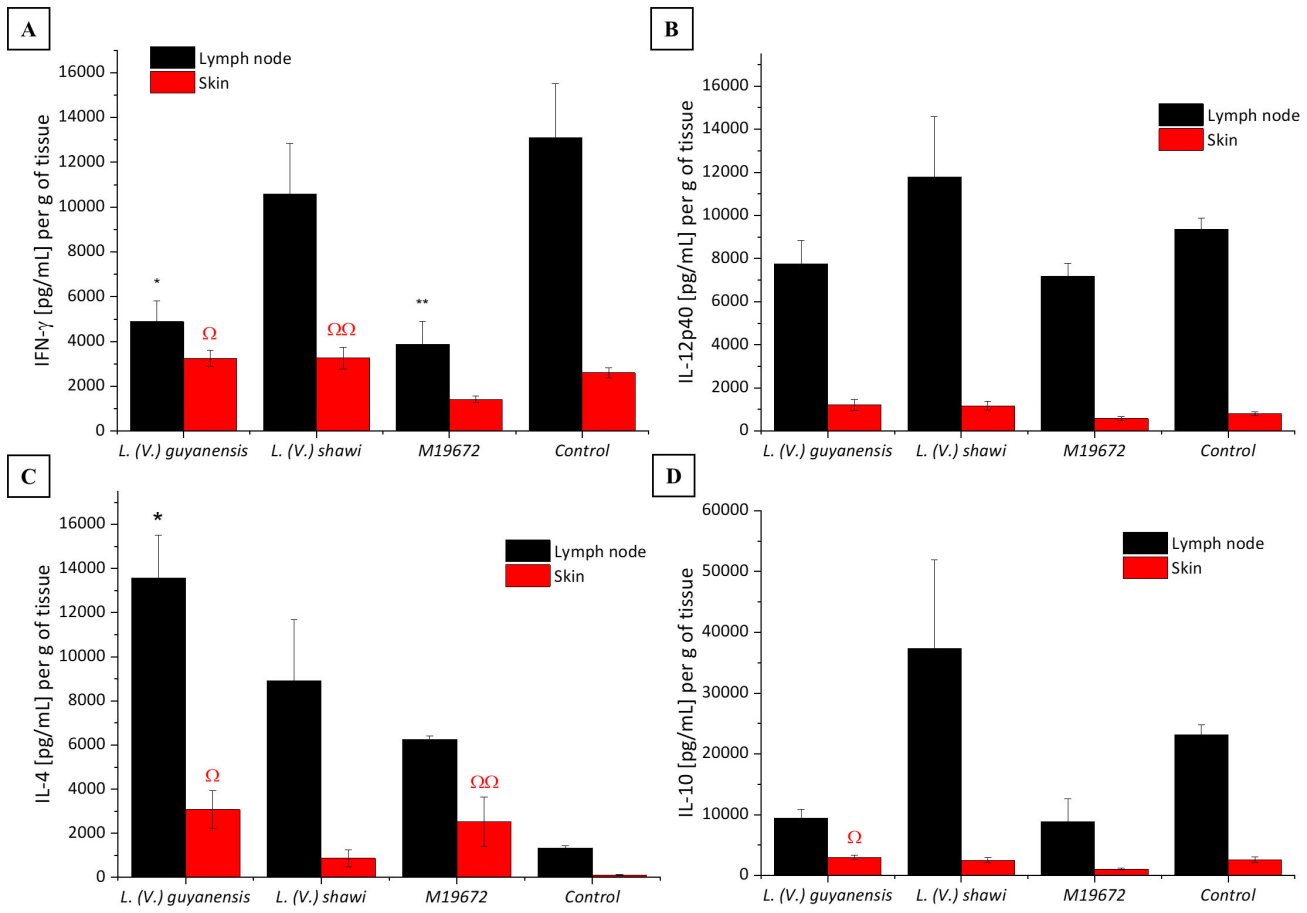
Quantitative analysis of cytokines in experimental BALB/c infection by different *Leishmania (Viannia)* species at 35 days PI. Data presented are the mean values ± standard error. **(A)** INF-γ production in skin and lymph node of BALB/c mice infected by hybrid strain M19672, *L. (V.) guyanensis* (M4147), *L. (V.) shawi* (M8408), and non-infected control group. **(B)** IL-12p40 production in skin and lymph node of BALB/c mice infected by hybrid strain M19672, *L.* (*V.*) *guyanensis* (M4147), *L. (V.) shawi* (M8408), and non-infected control group. **(C)** IL-4 production in skin and lymph node of BALB/c mice infected by hybrid strain M19672, *L. (V.) guyanensis*, *L. (V.) shawi*, and non-infected control group. **(D)** IL-10 production in skin and lymph node of BALB/c mice infected by hybrid strain M19672, *L.* (*V.*) *guyanensis* (M4147), *L.* (*V.*) *shawi* (M8408), and non-infected control group. Ω: p-value < 0.05 to *L. (V.) guyanensis* infection compared to M19672 infected group. ΩΩ p-value < 0.05 to *L. (V.) shawi* infection compared to M19672 infected group. *p-value < 0.05 to *L.* (*V.*) *guyanensis* infection compared to control group. **p-value < 0.05 to M19672 infection compared to control group.

The data on cytokine quantification in skin and lymph nodes of the experimental groups are summarized in **Table 1**. IFN-γ production in the skin and lymph nodes of mice infected with the hybrid parasite was significantly lower (1418 pg/mL/g and 3873 pg/mL/g, respectively; P < 0.05) than that detected in mice infected with either parental species, *L. (V.) guyanensis* (skin: 3232 pg/mL/g; lymph nodes: 4892 pg/mL/g) and *L. (V.) shawi* (skin: 3271 pg/mL/g; lymph nodes: 10,590 pg/mL/g) or in uninfected controls (skin: 2604 pg/mL/g; lymph nodes:13,090 pg/mL/g) (**Figure 4A**). In contrast, IL-4 levels were significantly elevated (P < 0.05) in the skin of hybrid-infected mice (2527 pg/mL/g) compared to the control group (107.7 pg/mL/g) (**Figure 4C**). Similarly, *L. (V.) guyanensis* infection induced higher IL-4 production in both the skin (3073 pg/mL/g) and lymph nodes (13,570 pg/mL/g) compared to uninfected mice (P < 0.05). IL-10 production in the skin of hybrid-infected mice (1061 pg/mL/g) was significantly lower than in the *L. (V.) guyanensis*-infected group (3003 pg/mL/g; P < 0.05). No significant differences were observed in IL-12p40 levels between the hybrid and parental infections (**Figures 4B, D**). Interestingly, IFN-γ levels in the skin of *L. (V.) guyanensis*-infected mice did not differ from those in uninfected controls (P > 0.05), although lymph node production was significantly reduced compared to controls (P < 0.05). Mice infected with *L. (V.) shawi* showed cytokine levels—both pro-inflammatory and anti-inflammatory comparable to those of uninfected controls in both analyzed tissues (P > 0.05) (**Figure 4**).

**Table 1:** Cytokine production (pg/mL/g tissue) in skin and lymph nodes of BALB/c mice infected with hybrid and parental *Leishmania* species or uninfected (control).

Cytokine	Tissue	*L. (V.) guyanensis*	*L. (V.) shawi*	M19672 (Hybrid strain)	Control
IL-4	Lymph Node	13570 ± 1949	8902 ± 2770	6251 ± 153.6	1330 ± 102.7
	Skin	3073 ± 874.4	871.5 ± 381.5	2527 ± 1122	107.7 ± 18.88
IFN-γ	Lymph Node	4892 ± 916.2	10590 ± 2251	3873 ± 1023	13090 ± 2428
	Skin	3238 ± 360.9	3271 ± 483.5	1418 ± 142.2	2604 ± 230.8
IL-12p40	Lymph Node	7764 ± 1070	11788 ± 2804	7189 ± 594.6	9352 ± 537.8
	Skin	1213 ± 256.8	1174 ± 202.7	577.8 ± 90.22	807.6 ± 84.94
IL-10	Lymph Node	9441 ± 1411	37357 ± 14539	8866 ± 3783	23107 ± 1694
	Skin	3003 ± 320.7	2509 ± 408.9	1061 ± 143.9	2578 ± 473

*Cytokine concentrations are presented as mean ± standard error of the mean (SEM) and expressed in pg/mL per gram of tissue. M19672 corresponds to the hybrid *Leishmania* isolate. Statistical significance is discussed in the results section.

## 4 Discussion

The aim of this study was to evaluate the infection behavior of the hybrid *L*. (*V*.) *guyanensis*/*L*. (*V*.) *shawi* (M19672) in relation to the parental species. Infection by the hybrid parasite was found to occur earlier and progress faster compared to the parental strains, which exhibited slower lesion development, peaking at later time points. The evolution of the swelling was quite heterogeneous when comparing the three groups of parasites, with *L*. (*V*.) *shawi* causing significantly less swelling than the other two strains. Notably, *L*. (*V*.) *guyanensis* produced lesions with a diameter more than double that of the hybrid parasite. These findings suggest that the inheritance of virulence factors from both *L*. (*V*.) *guyanensis* and *L*. (*V*.) *shawi* led to lesions displaying an intermediate profile compared to those observed in the parental species. These results show that a single recombination event—even between closely related parental species—can induce biological changes, underscoring the need for broader investigations of hybrid lineages within the *Leishmania* genus.

For the cytokine analysis, the 35^th^ day post-infection was chosen due to the difference in lesion profiles among the three groups (**Figure 1** green arrow), as well as the prognosis for disease progression or regression. Thus, pro-inflammatory cytokines IFN-γ and IL-12, as well as anti-inflammatory cytokines IL-4 and IL-10, were quantified in the skin and lymph nodes of BALB/c mice. Infection with the hybrid parasite resulted in higher IL-4 production in the skin compared to the control group. IL-4 is classically associated with the Th2-type cellular immune response, which is linked to disease progression in the classical *L.* (*L*.) *major* infection model (Hurdayal and Brombacher, 2014). However, no progressive lesion was observed; instead, it was suggestively in the process of healing. The role of IL-4 has been questioned following evidence that it stimulates dendritic cells from BALB/c mice to produce IL-12 in the presence of *Leishmania* (*Leishmania*) *major* during the early stages of infection (Biedermann et al., 2001). Other studies suggest that IL-4 can be expressed in contexts of *Leishmania* infection even in the absence of clinical lesions, both in animal models and in humans (Baratta-Masini et al., 2007; Felizardo et al., 2012). Notably, at 35 days post-infection, *L*. (*V*.) *guyanensis* elicited higher IL-10 production in the skin than did the hybrid parasite. The difference in IL-10 production may reflect the progressive stage of the swelling caused by *L*. (*V*.) *guyanensis* and the regressive stage of the swelling observed in the paws of mice infected with the hybrid parasite. When examining swelling progression in the skin of the groups infected with *L*. (*V*.) *guyanensis* and *L*. (*V*.) *shawi*, higher IL-4 production supports the findings, as it was quantified in greater amounts in the first group, which exhibited clear disease progression, while the second group showed more discrete progression of skin impairment. Additionally, the production of IFN-γ in the lymph nodes of mice infected with the hybrid parasite was lower than that observed in the uninfected control group. This finding was similar to what was observed in the infection with the parental species *L*. (*V*.) *guyanensis*. The high concentration of IL-4 associated with low levels of IFN-γ observed in the group infected with the hybrid parasite and *L*. (*V*.) *guyanensis* has been described in other studies; however, it is classically associated with a progressive infection scenario (Szabo et al., 1997; Borges et al., 2018). Intriguingly, the group infected with the hybrid parasite displayed a profile indicative of resolution at the site of the swelling while maintaining parasitism similar to that of the other groups until late stages of infection.

In infection with *L*. (*V*.) *guyanensis*, higher production of the cytokine IL-4 was observed both in the skin and in the draining lymph nodes compared to the uninfected control group. The promotion of a Th2 response by IL-4 has been associated with macrophage activation toward the polyamine and arginase pathways, favoring parasite persistence, as well as with the inhibition of IL-12 production, which would otherwise recruit Th1 cells to the infection site (Kropf et al., 2005). The data obtained from the experimental infection of BALB/c mice with *L*. (*V*.) *guyanensis* indicates a Th2-type response profile, with a predominance of IL-4 and the presence of IL-10, which apparently favored swelling progression during the chronic phase of the disease (Himmelrich et al., 1998). Th1-type cytokines did not show significant differences compared to uninfected mice in the skin. However, in the lymph nodes, the *L*. (*V*.) *guyanensis*-infected group showed lower IFN-γ production compared to the uninfected group. Although still underexplored, the infection profile of *L*. (*V*.) *guyanensis*, both in experimental models and human infections, has been described as more related to susceptibility (Martinez et al., 1991; Matta et al., 2009; Gomez et al., 2018). Infection experiments in susceptible murine models have shown that lesion progression in BALB/c mice peaks around the 5th week post-infection (Matta et al., 2009), which supports our findings.

During experimental infection with *L*. (*V*.) *shawi*, swelling progression remained limited until day 28 post-infection. By 35 days post-infection, slight paw swelling was observed; however, it did not progress to a typical lesion as seen with other species. Despite the characteristic infection control profile, there was a similar parasitic load to the other groups and no predominance of Th1-type cytokines in the skin of the mice.

The substantially higher parasite load values observed for the hybrid strain at the upper time limit of the experiment allowed for the selection of a linear model to describe its temporal dynamics. However, the trend curves—even based on log-transformed data—indicate that both parental strains exhibit a reduction in parasite load at some point between days 38 and 60 (corresponding to the peak and the upper time limit of the experiment, respectively). Integrating the statistical analysis results, a similar temporal pattern in parasite load dynamics could be observed between *L*. (*V*.) *shawi* and *L*. (*V*.) *guyanensis*, with a peak at day 38 followed by a decline toward day 60. In contrast, the hybrid strain exhibited a significantly higher parasite load compared to both parental strains and followed a distinct temporal pattern, with a continuous and significant increase up to day 60. These findings may indicate an adaptive advantage of the hybrids related to increased transmissibility. It is noteworthy that these parasites were isolated from patients with ATL in Santarém (PA), where they are part of the disease transmission cycle (Jennings et al., 2014).

A possible hypothesis is that *L*. (*V*.) *shawi* may remain silent in BALB/c mice due to the absence of virulence factors or possibly due to a tolerance induction pathway. This hypothesis could also account for the persistent parasitemia observed in mice infected with the hybrid parasite, despite the Th2-skewed cytokine profile in their skin. In the experimental infection model of C57BL/6 mice with *L*. (*L*.) *major*, parasite persistence in the host is associated with a population of regulatory T cells through IL-10-dependent or independent mechanisms (Belkaid et al., 2002). Further studies analyzing cytokines such as IL-5, IL-13, and regulatory T cell populations are needed to clarify the data obtained from BALB/c mice infected with *L*. (*V*.) *shawi*. Another important approach of investigation in this context would be the analysis of genes associated with virulence factors and their expression profiles among the parental species and the hybrid isolate M19672. It is important to note that experimental infection data for the parental species in BALB/c mice are scarce; consequently, our findings advance understanding of their behavior and provide a foundation for future studies.

In conclusion, the experimental infection model using *L*. (*V*.) *guyanensis*, *L*. (*V*.) *shawi*, and their hybrid in BALB/c mice revealed distinct infection profiles, with the hybrid lineage exhibiting intermediate virulence profile, and high parasite loads even in the absence of lesions.

Our findings highlight that a single genetic recombination event can substantially alter parasite biology, potentially affecting virulence and immune modulation. These results provide new insights into the complexity of host-parasite interactions in *Leishmania* infections and emphasize the need for further studies focusing on the role of hybridization in disease pathogenesis and parasite persistence.

## Data Availability

The raw data supporting the conclusions of this article will be made available by the authors, without undue reservation.
